# Can ChatGPT outperform a neurosurgical trainee? A prospective comparative study

**DOI:** 10.1080/02688697.2024.2308222

**Published:** 2024-02-02

**Authors:** Simon C. Williams, Joachim Starup-Hansen, Jonathan P. Funnell, John Gerrard Hanrahan, Alexandra Valetopoulou, Navneet Singh, Saurabh Sinha, William R. Muirhead, Hani J. Marcus

**Affiliations:** a Department of Neurosurgery, St George’s University Hospital, London, UK; b Wellcome/EPSRC Centre for Interventional and Surgical Sciences, University College London, London, UK; c Department of Neurosurgery, National Hospital for Neurology and Neurosurgery, London, UK; d Department of Neurosurgery, Imperial College Healthcare NHS Trust, London, UK; e Department of Neurosurgery, Sheffield Teaching Hospitals, Sheffield, UK

**Keywords:** Artificial intelligence, AI, natural language processing, large language model, ChatGPT, neurosurgery, healthcare

## Abstract

**Purpose:**

This study aimed to compare the performance of ChatGPT, a large language model (LLM), with human neurosurgical applicants in a neurosurgical national selection interview, to assess the potential of artificial intelligence (AI) and LLMs in healthcare and provide insights into their integration into the field.

**Methods:**

In a prospective comparative study, a set of neurosurgical national selection-style interview questions were asked to eight human participants and ChatGPT in an online interview. All participants were doctors currently practicing in the UK who had applied for a neurosurgical National Training Number. Interviews were recorded, anonymised, and scored by three neurosurgical consultants with experience as interviewers for national selection. Answers provided by ChatGPT were used as a template for a virtual interview. Interview transcripts were subsequently scored by neurosurgical consultants using criteria utilised in real national selection interviews. Overall interview score and subdomain scores were compared between human participants and ChatGPT.

**Results:**

For overall score, ChatGPT fell behind six human competitors and did not achieve a mean score higher than any individuals who achieved training positions. Several factors, including factual inaccuracies and deviations from expected structure and style may have contributed to ChatGPT's underperformance.

**Conclusions:**

LLMs such as ChatGPT have huge potential for integration in healthcare. However, this study emphasises the need for further development to address limitations and challenges. While LLMs have not surpassed human performance yet, collaboration between humans and AI systems holds promise for the future of healthcare.

## Introduction

The hype around artificial intelligence (AI) has been colossal, and its impact in healthcare is ever-increasing.^1^^,^^2^ The latest in a series of disruptive additions to the field is ‘ChatGPT’. ChatGPT (OpenAI, L.L.C., San Francisco, CA) is a natural language processing (NLP) generative AI, termed a large language model (LLM), that is able to draw upon vast datasets of text-based information in order to produce human-like responses to text based interactions.^3–5^ First released to the public in November 2022, ChatGPT experienced viral success – within five days of launch, ChatGPT had gained five million users,^6^ growing to 100 million by two months, making it the fastest growing consumer application in history.^7^ The hype was not unfounded – ChatGPT and its updated version (GPT 4.0), released in March 2023, demonstrated remarkable ability across a range of examinations, including ranking 90th centile in the Uniform Bar Exam, 89th in SAT Mathematics, and even achieving a score of 77% in advanced sommelier theory, demonstrating its breadth of applicability.^8^ Within months, the deluge of publications citing ChatGPT’s abilities in healthcare had begun, with applications in clinical workflow,^9–12^ education,^13–15^ and medical research.^3^ Despite valid concerns, it is clear that a disruptive technology has entered the world of healthcare.^3^

Evidently, AI models may assist with, and even eventually replace, numerous tasks currently performed by junior clinicians. Yet, the utility of ChatGPT in medical interview scenarios is one that is largely unexplored.^16^ Neurosurgery is one of the most competitive training specialties within the United Kingdom (255 applicants to 16 places nationally in 2022, resulting in a competition ratio of 1:16 for ST1 entry^17^). Neurosurgical interviews encompass a perfect setting to test the limitations of ChatGPT, given their notoriety for highly stressful situations in which applicants must draw upon a range of clinical knowledge, management strategies, and ethical considerations to synthesise appropriate and high-scoring answers.^18^ This integration of factual knowledge with decision-making capabilities is well-suited to AI performance – indeed, ChatGPT has already demonstrated its ability to answer neurosurgical examination questions.^19^

This study aimed to compare neurosurgical interview performance between ChatGPT and human applicants to the neurosurgical specialist training programme in the United Kingdom. In doing so, this study aims to provide further evidence regarding the advancement of AI and LLMs in healthcare, expanding on previous work demonstrating their utility in healthcare education.^16^ Further, this research aims to serve as a checkpoint for the ability of AI to emulate clinician performance.

## Methods

### Summary of methods

A set of neurosurgical national selection interview questions, encompassing clinical and management scenarios, were created and validated. Eight participants then sat the interview online, which were recorded for marking. The interview questions were used as prompts for ChatGPT, and the answers recorded. Audio transcripts of human and ChatGPT interviews were anonymised and sent to three neurosurgical consultants/attendings for marking (each with prior experience in national selection interviews). ChatGPT and human performance were then compared. Ethical approval was not required for this study in accordance with our local ethics department’s clause regarding research involving non-sensitive, completely anonymous educational tests, surveys, and interview procedures when the participants are not defined as ‘vulnerable’ and participation will not induce undue psychological stress or anxiety. This was confirmed with our local ethics department.

### Generation of interview questions

Neurosurgical training interviews in the UK currently consist of three stations: (1) ‘Curriculum Vitae (CV)/Understanding of Specialty’ – dissecting the applicants clinical, academic, and educational performance to date, and assessing the applicant’s knowledge of current ‘hot topics’ pertaining to neurosurgery; (2) ‘Clinical Scenario’ focusing on technical knowledge and problem-solving skills concerning common neurosurgical clinical scenarios; and (3) ‘Management’ – focusing on the applicants performance in managing stressful clinical, ethical, and managerial situations that a neurosurgical trainee may encounter.

Given the lack of applicability of ‘CV’ based questions for the AI, this station was excluded from our study. A single ‘Clinical’ and ‘Management’ station were created. Interview stems and questions were devised by three neurosurgical trainees with recent experience of sitting national selection interviews (SW, JPF, and JGH). Interview questions then underwent a two-step validation process. First, three neurosurgical consultants with experience in scoring neurosurgical national selection interviews (NS, SS, and HJM) were asked to answer the following questions for each proposed interview question: *‘Do you agree that this question is typical of a neurosurgical national selection interview question’*. Second, interview participants answered the same question following their interview. Responses were recorded using a five-point Likert scale (1 = strongly disagree; 2 = disagree; 3 = neither agree nor disagree; 4 = agree; 5 = strongly agree). A freetext box was also present for each question to enable specific comments. The Likert scale was designed in accordance with existing recommendations.^20^ Questions with median agreement of <3 were excluded from the study. Through free-text analysis, question stems underwent a series of iterative changes until a consensus was reached. Interview questions and median agreement for the final set of questions for both clinical and management scenarios are shown in Table 1.

**Table 1.** Clinical and management scenario interview questions validation by consultants and participants.

	Consultant agreement (Med + IQR)	Participant agreement (Med + IQR)
*Clinical scenario*		
You are the ST1 neurosurgical doctor covering the department with your neurosurgical senior registrar, who has asked you to hold the on-call phone whilst she is in theatre. You receive a phone call from an A&E consultant in a nearby hospital who wants to urgently transfer a 28-year-old man who was in a road traffic accident and has an extradural haematoma. How would you proceed?	4 (4–5)	5 (4–5)
The patients CT head scan is sent over to your computer. It shows a large, hyperdense, convex lesion overlying the left cerebral hemisphere. What are your differentials?	4 (3–5)	4 (2–5)
You arrange for the case to be urgently transferred to the hospital, when you receive a bleep from the bed manager explaining that there are no beds, and that there is no capacity for the patient. How would you proceed?	5 (4–5)	5 (5–5)
The patient arrives after 45 minutes. On arrival, you examine the patient and find that the patient opens his eyes to painful stimuli, makes noises but no discernible words, and withdraws from pain but does not localise. What is his GCS?	4 (2–5)	5 (3–5)
On further examination, the patient’s left pupil is dilated and unreactive to light. How would you proceed?	5 (4–5)	5 (5–5)
The patient undergoes a left sided craniotomy and evacuation of an extradural haematoma. His left pupil is reactive post-op. The patient is managed in intensive care post-operatively. Please describe the management of intracranial pressure in the intensive care setting.	5 (4–5)	5 (5–5)
What is the Monro–Kellie hypothesis?	5 (2–5)	5 (3–5)
*Management scenario*		
You are the neurosurgical ST1 on a night shift. You have noticed that your registrar has been taking medication during a shift. She tells you that she has had a difficult time sleeping recently, and that her GP prescribed her a short course of diazepam, but that she has received approval from the night consultant to work. How would you approach this situation?	5 (4–5)	5 (5–5)
As you are discussing this with her, you receive three bleeps. (1) A drug chart error that needs re-writing. (2) A patient’s external ventricular drain has stopped draining. (3) The acute subdural admission has arrived. How would you proceed?	5 (3–5)	4 (4–5)
You quickly amend the drug chart, and are assured that the EVD patient is GCS 15. You then go to see the subdural patient and prepare him for theatre. As you clerk the patient you notice that he has a positive swab result for Covid-19. How would you proceed?	5 (4–5)	5 (4–5)
The patient is taken to theatre. Your registrar is scrubbed and ready, but you notice she is swaying. When asked if she is okay she mumbles that ‘she is fine’, and that the consultant said it was fine for her to continue. How would you approach this situation?	5 (4–5)	5 (5–5)
You go to call the consultant and the registrar grabs the phone out your hand, tells you to mind your own business, and tells the theatre staff that she is ready to start the operation now. How would you proceed?	5 (4–5)	5 (5–5)
How do you recognise that you’re stressed?	3 (2–5)	5 (4–5)

Consultants and participants were asked ‘Do you agree that this question is typical of a neurosurgical national selection interview question’. Responses were recorded using a five-point Likert scale (1 = strongly disagree; 2 = disagree; 3 = neither agree nor disagree; 4 = agree; 5 = strongly agree).

### Participant selection

Eight human participants were selected to undergo interview. Participants were initially identified from a National Neurosurgical Teaching Course – Fundamentals for Early Neurosurgical Trainees (FENT), with subsequent participant additions identified through snowball sampling methodology. Participants were all doctors currently practising in the UK who had applied for a neurosurgical National Training Number during the 2022–2023 application.

### Data collection

Structured interviews were conducted over Zoom (Zoom Video Communications, Inc., San Jose, CA), with 10 minutes allocated for each station. Participants were informed of the study details and provided verbal consent for their data to be used for research purposes. Audio recordings were made of each interview for later analysis. All interviews were conducted within a four-week period of real-life neurosurgical ST1 interviews (March 2023).

ChatGPT interview answers were derived using ChatGPT (Version 3.5) in January 2023. Text inputs for each question are as shown in Table 1. ChatGPT answers to both clinical and management scenarios are provided in Appendix 1. An audio recording was made by the authors using ChatGPT’s answers as an exemplar.

Audio recordings (both human participants and ChatGPT recording) were anonymised using a voice anonymising software (iMovie version 10.2.5, Apple, Cupertino, CA) and distributed to three neurosurgical consultants with experience in scoring neurosurgical ST1 interviews. Interviews were scored in triplicate in a blinded fashion. Scoring criteria was identical to the existing criteria used in national selection interviews, using the criteria outlined in Table 2, along with free-text responses. A total score out of 30 was calculated for each scenario, and an overall score out of 60 for complete performance. Finally, we captured whether participants achieved a neurosurgical ST1 training number in real-world interviews, to enable benchmarking of AI performance.

**Table 2.** Marking criteria for interview audio recordings.

*Clinical scenario*	
Technical knowledge and clinical expertise	/10
Problem solving skills	/10
Communication skills	/10
*Management scenario*	
Judgement under pressure	/10
Problem solving skills	/10
Professional Integrity	/10
*Overall assessment of performance*	
Comments:	

A total score out of 30 was calculated for each scenario, and an overall score out of 60 for complete performance.

### Data analysis

Results are presented descriptively, showing overall performance and rank of candidate and ChatGPT performance. Inferential statistics were used to compare performance between human and AI performance. Mann–Whitney’s *U*-test was used to compare scores, with *p* < .05 deemed significant. Data were tabulated using Microsoft Excel (Microsoft, Redmond, WA) and statistical analysis was performed using GraphPad (Prism Version 9, GraphPad Software Inc., La Jolla, CA).

## Results

Interviews for eight human interviews and one ChatGPT interview were scored. The interview transcript for ChatGPT can be found in its entirety in Appendix 1. All interviews were scored across six subdomains. Baseline data for human participants are shown in Table 3.

**Table 3.** Baseline characteristics of human participants.

Gender M:F	6:2
Age (median + IQR)	26.5 (IQR 26–30)
Current role	
Junior clinical fellow	3
Foundation year two	3
Higher education	2
Number of neurosurgical applications (total, including this current year’s application)	
1 application	4
2 applications	4

**Figure 1.**
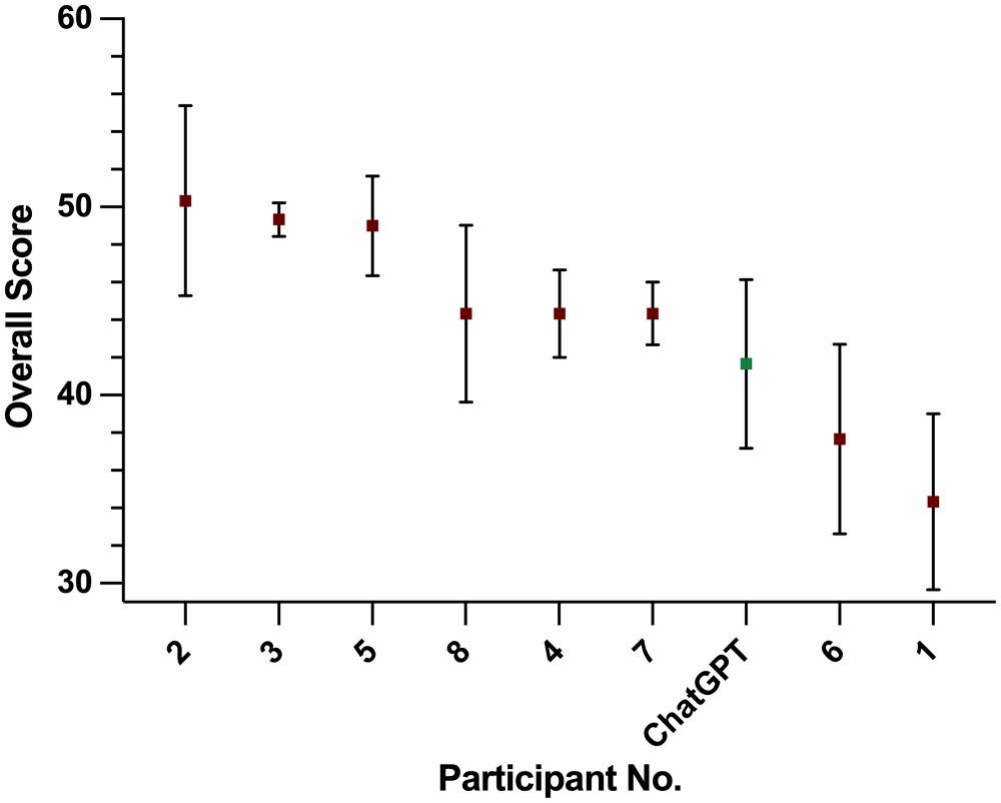
Overall interview score for human and ChatGPT interviews (mean and standard error). Participants were scored out of 10 for three subdomains within both clinical and management scenarios, giving a total score out of 30 for each scenario, and a combined overall score out of 60. Interviews were marked by three neurosurgical consultants in a blinded fashion.

Overall interview score for human and ChatGPT interviews are shown in Figure 1. Mean overall score for ChatGPT’s interview was 41.7/60 (SE 4.5, 69.5% overall), ranking seventh out of nine participants. The maximum overall score was achieved by participant 2, with a mean score of 50.3 (SE 5.0, 82.2%), and the minimum score by participant 1 (mean 34.3, SE 4.7, 57.2%).

Individual analysis of subdomains reveals scores consistent with overall scores, and combined subdomain analysis. The top performers (participants 2, 3, and 5) consistently scored highest across *Knowledge, Problem Solving, Communication, Judgement,* and *Integrity* subdomains. A similar trend was seen for the lower ranking participants. ChatGPT ranked seventh out of nine across all categories, except communication for which it ranked sixth.

**Figure 2.**
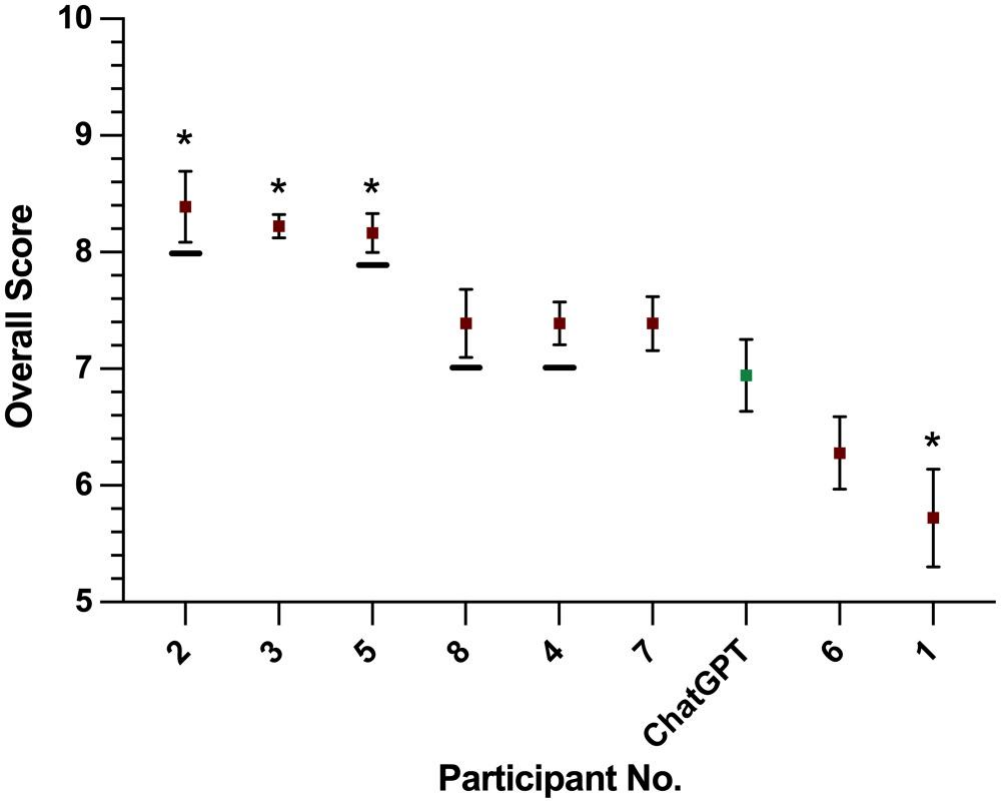
Combined subdomain score analysis for human and ChatGPT interviews (mean and standard error). Individual subdomains can be found in Table 2; each subdomain was scored out of 10. Asterisks denote a statistically significant difference (*p* < .05, Mann–Whitney’s *U*-test) when compared to ChatGPT’s score. Underlines indicate participants who achieved an ST1 national selection number in real life. Three participants (participants 2, 3, and 5) scored statistically significantly better than ChatGPT, whilst participant 1’s score was worse. There was no significant difference between four human interview subdomain scores and ChatGPT’s.

Combined analysis across scores of all subdomains (i.e. problem solving skills, professional integrity, see Table 2, each ranked out of 10) reveals a mean subdomain score for ChatGPT of 6.9/10 (SE 0.3), ranking seventh out of nine participants (Figure 2).

Three participants (participant 2 (mean 8.4, SE 0.3), participant 3 (mean 8.2, SE 0.1), and participant 5 (mean 8.2, SE 0.2)) scored statistically significantly greater scores than the ChatGPT model (Mann–Whitney’s *U*-test, *p* < .05). ChatGPT performed significantly better than one human participant (participant 1, mean 5.7, SE 0.4). All four participants who went on to achieve a neurosurgical ST1 post (participants 2, 4, 5, and 8) scored higher than ChatGPT. Comparable scores were found between ChatGPT and four participants (participants 4, 6, 7, and 8), ranging from 7.4 to 6.2, with ChatGPT scoring 6.9. No significant difference was found in subdomain scores between these four participants and ChatGPT.

Analysis of free-text comments for each participant revealed critiques of ChatGPT’s performance. Notably, all three markers commented on ChatGPT calculating the Glasgow Coma Scale score incorrectly. One consultant highlighted that ChatGPT did not respond in the stereotyped ‘A to E’ approach to stabilising a patient, prior to further management. Furthermore, the structure of ChatGPT’s answers were called into question, as one consultant highlighted *‘[Participant] didn’t say patient safety first’.* Another consultant highlighted the vagueness of ChatGPT’s answers, particularly when asked to define the Monro Kellie Doctrine, and explain intensive care management of neurosurgical patients – *‘…answers vague, non-specific…didn’t really get to the crunch of the answer’.*

## Discussion

### Principal findings

In recent years, the incorporation of AI technologies in healthcare has shown great promise in enhancing efficiency, accuracy, and accessibility of medical services.^3^ Amongst the positive expectation, however, the transformative potential of AI has also given rise to concerns and anxieties amongst clinicians.^21^^,^^22^ Fear of potential job displacement and the diminishing role of human expertise has led to apprehensions about the future of healthcare practice. In 1950, Alan Turing devised his famous Imitation Game as a litmus test of machine intelligence – poised to differentiate humans from machines.^23^ In this small study, we run a Turing Test of sorts, through comparison of human neurosurgical applicants with ChatGPT in a typical neurosurgical national selection style interview, generating several key findings.

Whilst ChatGPT performed comparatively with many participants, it scored lower than all participants who went on to attain neurosurgical training posts. In simple rank, ChatGPT fell behind six human competitors. This result was in contrast to expected performance, given the detail and quality of ChatGPT’s transcript answers. There are several possible reasons for this.

First, ChatGPT was objectively incorrect when answering certain factual questions. The incorrect scoring of the Glasgow Coma Scale score, for example, was picked up upon by all examiners. ChatGPT was asked *‘…the patient opens his eyes to painful stimuli, makes noises but no discernible words, and withdraws from pain but does not localise. What is his GCS?’*, to which ChatGPT responded that the patient was E1V2M3, as opposed to the correct E2V2M4. Accurate and rapid calculation of the GCS is a skill expected of neurosurgical applicants, and we anticipated that this element of the interview would be rapidly calculable by ChatGPT given its discrete and objective nature. The generation of factual inaccuracies presented in a confident manner is a key concern regarding generative AI models (known as hallucinations).^3^^,^^24^ These factual discrepancies are far from trivial given the breakdown of GCS is vital in informing neurosurgical decision making.

Second, several of the criticisms of the ChatGPT interview were based upon structure and style of answer. *‘I would first assess the patient using an A–E approach’* is a sentence dogmatically ingrained within medical graduates, and serves to highlight a set of unspoken rules amongst which clinical interview candidates abide, the absence of which mark ChatGPT out. This demonstrates a critical limitation of AI in healthcare settings – and that is the fundamental attribute of humans to attribute intelligence not only to content, but also delivery. Structure, style, and unwritten rules are all crucial to human performance, and indeed all those who went on to achieve neurosurgical training numbers scored highly in communication. Further training using model datasets may improve ChatGPT’s ability to mimic the clinical interviewee, and ultimately come across as ‘more human’.

However, ChatGPT's performance is not entirely discouraging. Our data show that ChatGPT performed comparably with four participants, two of whom went on to achieve neurosurgical numbers. Neurosurgical training positions, both in the UK and abroad,^17^^,^^25^ are highly sought after and typically attract high-performing candidates. The comparative performance of ChatGPT with some human participants signals a paradigm shift in the ability of LLMs in healthcare, suggesting that ChatGPT is highly relevant in the field, whilst showcasing the need for incremental improvements in AI technology before it can meaningfully match human doctors.

### Comparison to current literature

ChatGPT has been trialled in several neurosurgical settings thus far. Hopkins *et al.* describe the application of ChatGPT in answering written neurosurgical questions.^19^ ChatGPT answered 60.2% of questions correctly, performing comparably with two residents (registrar level) (mean overall score 61.5%), and comparably with the IF index of the question bank – well within the upper and lower quintiles of 72% and 51%, respectively.^19^ The authors noted that hallucinations and ‘best guesses’ were a key limitation of ChatGPT.^19^ Ali *et al.* performed a similar comparative study, again demonstrating the comparative performance (but not surpassing) of ChatGPT3.5 with humans in both ‘single best answer’ and ‘multiple choice’ neurosurgical questions.^26^ Both these studies’ findings mimic our own. Ali *et al.* went one step further in trialling ChatGPT4.0, the latest version of the generative AI, and noted superior performance compared to human counterparts.^26^

So, should we fear the creep of AI? At present, and based upon the results of our small study, the prospect of AI replacing human doctors in the near future seems unlikely. Instead, it is more probable that AI, including ChatGPT, will be utilised as a valuable decision support tool, assisting healthcare professionals in their day-to-day roles. AI may be integrated into electronic healthcare systems in order to act as a virtual assistant, enabling shared decision making and increased efficiency.^24^ Indeed, many healthcare systems have already invested heavily in NLP integration.^27–29^ Further, the use of LLMs in medical education and interview preparation is likely to expand. Opinions are divided as to whether AI will enhance access to high-level education or will deepen existing inequalities.^30^ Evidently, prior to wider uptake in healthcare, LLMs such as ChatGPT have a range of issues to overcome, including moderating risk of bias and transparency issues, and answering key questions regarding ethical and equitable use.^3^

### Strengths and limitations

Our study has several strengths and limitations. We ensured that our interview questions accurately represented real neurosurgical interviews through input from recent interviewees and validation by consultants and applicants. Second, our candidate selection was robust and involved current neurosurgical applicants. Participants were interviewed around real-life national selection interviews to ensure peak preparation. A limitation of our study is the lack of radiological image interpretation, such as cranial or spinal imaging. Selection bias may have been introduced by recruiting via a neurosurgical teaching course. We acknowledge that the performance of ChatGPT’s transcript by a human adds a confounding factor in performance. Our justification for this is twofold; first, if we had chosen to provide transcripts only of all interviews (i.e. not audio recordings), a core facet of interview performance would have been lost; and second, transcripts derived from ChatGPT and human recordings are necessarily and noticeably different – syntactic and semantic differences between ChatGPT and human transcripts would have resulted in recognition of the AI transcript, ultimately unblinding the study. Finally, whilst our team used the latest version of ChatGPT (version 3.5) at the time, an updated version has since been released (version 4.0), which has demonstrated superior ability in examinations.^8^

## Conclusions

This study compared the performance of ChatGPT, an AI LLM, with human neurosurgical applicants in a neurosurgical national selection style interview. ChatGPT scored lower than all participants who went on to achieve a neurosurgical training post, signalling that AI capabilities do not currently surpass human levels of performance. Iterative improvements in LLMs are required in order to make the meaningful impact in healthcare that the technology promises.

However, comparable performance of ChatGPT with some human participants in this study underlines the potential for AI as a virtual assistant, enabling shared decision making and increased efficiency. Future developments should address limitations and foster collaboration between humans and AI systems.

## Data Availability

The data that support the findings of this study are available from the corresponding author, [Simon C. Williams], upon reasonable request.

## Appendix 1. Transcribed ChatGPT and selected participant answers

Participant answers have been transcribed verbatim. Individual participant numbers have not been provided to protect the anonymity of participants. However, and indication of marking performance has been provided.

(1) Clinical station

**You are the ST1 neurosurgical doctor covering the department with your neurosurgical senior registrar, who has asked you to hold the on-call phone whilst she is in theatre. You receive a phone call from an A&E consultant in a nearby hospital who wants to urgently transfer a 28 year old man who was involved in a road traffic accident and has an extradural haematoma. How would you proceed?**

(a) ChatGPT’s response

I would proceed as follows:

- Confirm the patient’s vital signs and other relevant details such as Glasgow Coma Scale score, vital signs, and any pre-existing medical conditions.
- Assess the urgency of the transfer and allocate the necessary resources.
- Consult with my senior registrar, if available, and other members of the neurosurgical team to determine the best course of action.
- Arrange for the patient to be transferred to our hospital, ensuring that the necessary equipment and personnel are available.
- Prepare the operating room and team for the patient’s arrival, and ensure that the patient is taken to the operating room as soon as possible.
- Perform a thorough examination of the patient, including a CT scan, to determine the extent of the injury and plan the appropriate surgical intervention.
- Work with the rest of the team to provide life-saving treatment, including surgery if necessary.
- Keep the referring hospital informed of the patient’s condition and progress.

(b) Selected participant response

- ‘The first thing I would do is get the name and grade of the contact – the person that’s contacting me as well as their number in case we’re disconnected.
- I’d then want to establish more details about the patient, particularly the name, the age, and date of birth.
- I'd then want to establish the nature of the events. So I’d want to know the current haemodynamic status, whether they completed a primary survey according to ATLS principles, and what was found, as well as a secondary survey. I’d want to have an understanding of the haemodynamic status as well as a focused neurological exam including GCS, pupils, and lateralising neurology.
- I would then want to establish an ample history, particularly history of presenting complaint, allergies, the medications, when they last ate, and the events leading up to the accident. In particular, I’m interested in any antiplatelet or anticoagulant medications that the patient might be on.
- I’d then want to see the scans ideally, so I want to transfer the scans so I can have a look at them over the phone.’

*This participant scored well in knowledge & clinical expertise, problem solving, and in communication, and outperformed ChatGPT across the clinical interview scenario.*

**(1) The patients CT head scan is sent over to your computer. It shows a large, hyperdense, convex lesion overlying the left cerebral hemisphere. What are your differentials?**

(a) ChatGPT’s response

Given the description of the patient’s CT head scan, my differentials would be:

- Extradural hematoma
- Subdural hematoma
- Intracerebral hematoma
- Contusion
- Hemorrhagic stroke
- Neoplasm
- Abscess

It is important to consider the clinical context and additional information from the patient’s history and examination to arrive at a more specific diagnosis. Further imaging and diagnostic tests may also be required to confirm the diagnosis.

(b) Selected participants response

So my top differential is a left extradural haematoma. Also in my differentials is an acute subdural haematoma. These would be my main differentials. I’d also be worried about a fracture of the skull or a subgroup of haematoma on that left side. There may also be a degree of cerebral contusion or traumatic subarachnoid haemorrhage on that side. I’d want to look at the scan to confirm as well.

*This participant scored highly across the clinical station.*

Convex lesion…okay, and its causing mass effect. Okay, so it sounds like a subdural, an acute subdural haematoma with mass effect rather than an extradural.

*This participant scored comparatively poorly across the clinical station.*

**(1) You arrange for the case to be urgently transferred to the hospital, when you receive a bleep from the bed manager explaining that there are no beds, and that there is no capacity for the patient. How would you proceed?**

(a) ChatGPT’s response

I would proceed as follows:

- Confirm the current bed status with the bed manager and understand the reasons for the lack of capacity.
- Re-assess the urgency of the patient’s transfer and determine if alternative arrangements can be made to ensure the patient receives appropriate care.
- Consult with the referring hospital to determine if the patient can be stabilized and managed there until a bed becomes available.
- Consider transferring the patient to another hospital with available capacity, ensuring that proper transfer protocols and procedures are followed.
- If the patient’s condition is unstable and a bed is not immediately available, consider transferring the patient to the operating room for emergency surgery, if indicated.
- Keep the referring hospital, the bed manager, and the patient’s family informed of the situation and any changes to the patient’s status.
- Continuously reassess the patient’s condition and work with the rest of the team to ensure the patient receives appropriate and timely care.

(b) Selected participants response

So I would discuss with my senior if possible, um, there is a local ambulance service that can look if the patient can be transferred to another neurosurgical unit. I’d advise the A&E department to contact the nearest other neurosurgical unit to accept the patient.

*This participant performed worse when compared with ChatGPT.*

So at this point we’ve got a number of issues, first there is the patient safety issue, but I’ve also got the issue that theres no beds, so a resource issue. In the first instance, I’d continue with having the patient transferred over at the moment. The options are:

- We could potentially step somebody down from ITU or HDU, if the patient requires a HDU bed.
- We could look to discharge patients.
- The other possibility is we could treat them and have them stay in overnight recovery, until we find a bed for them.
- Then the other two options which would be less than ideal will be to bring the patient over, treat the life-threatening injury, and then transfer them to another neurosurgical unit, or…
- …potentially, have them transferred to a different neurosurgical unit altogether for management of their condition.

The first two options would be more ideal.

*This participant scored highly and outperformed ChatGPT.*

**(1) The patient arrives after 45 minutes. On arrival, you examine the patient and find that the patient opens his eyes to painful stimuli, makes noises but no discernible words, and withdraws from pain but does not localise. What is his GCS?**

(a) ChatGPT’s response

The patient’s Glasgow Coma Scale (GCS) score would be:

Eye opening response: 1 (opens to painful stimuli) Verbal response: 2 (makes noises but no discernible words) Motor response: 3 (withdraws from pain but does not localize)

So the patient’s GCS score would be 1 + 2 + 3 = 6.

A GCS score of 6 indicates a moderate to severe traumatic brain injury and requires immediate medical attention and close monitoring. The patient’s condition may rapidly deteriorate, and prompt neurosurgical intervention may be required to prevent further injury and improve outcomes.

(b) Selected participants response

So its, opens eyes to pain so its E2, making sounds so V2, and withdrawing so M4, so it’s a total score of 8. At this point I’m concerned about the patient’s airway so I’d re-assess the patient and ensure my seniors are aware.

So he has a GCS of 8, so this is a potential concern for… no… they have a GCS of 9, and I would like to progress to an A-E assessment and check that they still have airway patency, and if not I would like anaesthetic support and would put out an emergency call.

**(1) On further examination, the patient’s left pupil is dilated and unreactive to light. How would you proceed?**

(a) ChatGPT’s response

Given the finding of a dilated and unreactive left pupil, I would proceed as follows:

- Re-assess the patient’s Glasgow Coma Scale (GCS) score, taking into account the change in pupillary response.
- Notify the rest of the neurosurgical team and arrange for an urgent CT scan of the patient’s head to be performed.
- Continuously monitor the patient’s vital signs and neurological status, paying close attention to any changes that may indicate a deterioration in their condition.
- Consider administering mannitol or other agents to reduce intracranial pressure, if indicated.
- Prepare for emergency surgery, if necessary, and work with the rest of the team to ensure that the patient receives appropriate and timely care.
- Discuss the patient’s condition and the need for surgery with the patient’s family and obtain informed consent, if necessary.
- Continuously re-evaluate the patient’s condition and adjust the management plan as needed based on the patient’s response to treatment and the results of imaging and other diagnostic tests.

(b) Selected participants response

So I’m concerned about a raise in ICP and uncal herniation. I want to commence prompt management of ICP, including:

- Giving osmotic therapy, mannitol, 1 g/kilo;
- Make sure the head of the bed is at 30 degrees;
- Remove any collars;
- Making sure to have normothermia and normoxia, if the patient is intubated and ventilated we can aim for PaCO_2_ of 4.5–5.
- Id then also want to reassess the patient using an A–E approach to ensure airway patency, respiratory function, and cardiac stability, and then re-assess his neurological status,
- And then obviously I would like to make sure my seniors are aware, including on-call registrar and neurosurgical consultant.
- Finally, I’d want to make sure the theatre staff and theatre coordinator staff are aware and make sure relevant bloods and investigations were sent, and that the patient was booked on the emergency list. The patient will require a consent form 4, and I’d also inform the next of kin.

*This participant scored highly.*

Okay. So I’m worried about herniation. In this patient I would, what would I do, I would:

- Begin medical management of ICP.
- Make sure he is intubated and adequately sedated.
- I would give mannitol or hypertonic saline with the help of the anaesthetic team…
- …and then he needs urgent transfer to theatre for evacuation of the haematoma.
- Again, I would talk to my consultant or registrar prior to doing this. We may also put in an ICP wire at the same time.’

*This participant scored poorly in the clinical station.*

**(1) The patient undergoes a left sided craniotomy and evacuation of an extradural haematoma. His left pupil is reactive post-op. The patient is managed in intensive care post-operatively. Please describe the management of intracranial pressure in the intensive care setting.**

(a) ChatGPT’s response

The management of intracranial pressure (ICP) in the intensive care setting after a craniotomy for extradural hematoma evacuation typically involves the following:

- *Monitoring*: Continuously monitor ICP using a pressure-monitoring device such as an intraventricular catheter or a continuous lumbar drainage system.
- *Head of bed elevation*: Maintain the patient’s head in a slightly elevated position (30–45°) to reduce ICP and improve cerebral perfusion.
- *Sedation and paralysis*: Administer sedatives and neuromuscular blocking agents as needed to control agitation and prevent excessive movement, which can increase ICP.
- *Hyperosmolar therapy*: Administer mannitol or other hyperosmolar agents as needed to reduce ICP by decreasing cerebral edema.
- *Ventilation management*: Adjust the patient’s ventilation settings to maintain a normal PaCO_2_ and prevent hypercapnia, which can increase ICP.
- *Fluid management*: Avoid over-hydration and maintain a strict fluid balance to prevent cerebral edema and increase in ICP.
- *Nutrition*: Provide adequate calorie and protein intake to support brain recovery and minimize the risk of secondary brain injury.
- *Seizure prophylaxis*: Administer anticonvulsants as indicated to prevent seizures, which can increase ICP.
- *Re-evaluation*: Continuously re-evaluate the patient’s ICP, neurological status, and response to treatment, and adjust the management plan as needed.

The management of ICP in the intensive care setting is a complex and dynamic process that requires close monitoring and adjustment of multiple interrelated factors to ensure optimal outcomes for the patient.

(b) Selected participants response

Okay, so we’ve touched on a few of these topics already, but in the intensive care setting what we want to do is try to:

- Optimise pain analgesia and temperature to avoid anything that might agitate the patient.
- We want to keep the appropriately sedated to try and reduce any agitation again.
- Bed elevation to thirty degrees.
- We have the option of mannitol or hypertonic saline as well.
- We could try to hyperventilate the patient should we want to, maintaining a CO_2_ of 4 to 4.5 in the acute setting.
- If we needed to at the very end, we could consider CSF diversion using an EVD, or thiopentone based barbiturate coma, although this is far down the line. Alternatively, there is also cooling.

*This participant scored equivalently with ChatGPT.*

**(1) What is the Monro–Kellie hypothesis?**

(a) ChatGPT’s response

The Monro–Kellie hypothesis is a medical principle that explains the relationship between intracranial contents and intracranial pressure (ICP). The hypothesis states that the volume of the cranial contents, including the brain, blood, and cerebrospinal fluid (CSF), is constant and cannot be increased. Therefore, an increase in the volume of one component will result in a compensatory decrease in the volume of another component.

According to the Monro–Kellie hypothesis, an increase in ICP can result from an increase in any one of the following components:

- *Brain tissue volume*: An increase in brain swelling or edema can result in an increase in ICP.
- *Blood volume*: An increase in the volume of blood within the cranial cavity, such as from a subdural hematoma, can result in an increase in ICP.
- *Cerebrospinal fluid volume*: An increase in the volume of CSF within the cranial cavity, such as from hydrocephalus, can result in an increase in ICP.

The Monro–Kellie hypothesis is a fundamental principle in the management of increased ICP, and highlights the need to manage the volume of all components within the cranial cavity in order to prevent and control ICP elevations.

(b) Selected participants response

The Monroe-Kellie Doctrine states that the cranium has a fixed volume, and is made up of three constituents, brain, blood, and CSF. The body will try to compensate and protect the brain as much as possible by shifting out CSF and blood to try to maintain a stable intracranial pressure. However, at a certain point in time there’s no more CSF or blood to be displaced, and at that point you begin to see herniation syndromes, whereby the brain is pushed out of the skull to try and maintain the same volume.

*This participant scored equivalently with ChatGPT.*

*Management Station 1*

**(1) You are the neurosurgical ST1 on a night shift. You have noticed that your registrar has been taking medication during a shift. She tells you that she has had a difficult time sleeping recently, and that her GP prescribed her a short course of diazepam, but that she has received approval from the night consultant to work. How would you approach this situation?**

(a) ChatGPT’s response

You should approach the situation with a focus on patient safety and ethical considerations. Here are some steps you could take:

- Confirm the registrar’s claim of approval from the consultant.
- Assess the registrar’s cognitive and physical abilities to perform their duties safely.
- If necessary, consult with the consultant or another senior colleague to determine the best course of action.
- If there are concerns about the registrar’s ability to perform their duties safely, consider relieving them of their duties for the shift.
- Document the situation and any actions taken, including the reason for the registrar taking medication and the assessment of their ability to work safely.

Remember, the well-being of patients and the integrity of the medical profession should be the top priority in any decision made in this situation.

(b) Selected participants response:

So we’ve got a number of issues here.

- First, I've got a patient safety issue.
- Second, there is a colleague welfare issue. I've got a colleague that may be impaired to work since she has taken the medication, and I’d ultimately want to make sure they are well supported.
- Third, we’ve got a system issue. If the colleague is impaired to work then we need to find appropriate cover for her.

So in the first instance I’d:

- Talk to the colleague, highlight my concerns. I’m aware the consultant has said its okay for them to work, but, you know, given the fact they’ve had poor sleep and they’re on medication that can affect them it may be best that they go home and rest.
- Once I’ve had an open, non-confrontational conversation about this I can involve seniors as well. At this point, I could ascertain whether our seniors felt if it was safe for the registrar to continue.
- If it were determined that they weren’t, the on-call consultant would likely need to step down and provide cover. Meanwhile, I would contact the site manager to see if there is any other emergency cover that we could provide.
- In the meantime, I’d be happy to take up some of the duties which I am confident to do.’

*This participant scored highly.*

So this is a colleague that sounds like she’s struggling, and I would just check in with her and be sympathetic and ask her if she feels okay and safe to work. I suppose my concern with diazepam is that it can make you drowsy. I would just clarify that the consultant is aware that she on shift, and if this has been cleared with occupational health. I would also ask if there is anything that I can do specifically to help her during the night shift. But essentially, I’d gather information as to whether she’s safe to work here.

*This participant scored an average score across the management station.*

**(1) As you are discussing this with her, you receive three bleeps. 1) a drug chart error that needs re-writing. 2) a patient’s external ventricular drain has stopped draining. 3) the acute subdural admission has arrived. How would you proceed?**

(a) ChatGPT’s response

In this situation, you should prioritize the bleeps based on the urgency of the situation and delegate tasks as appropriate. Here are some steps you could take:

- Assess the urgency of each bleep:
  - The external ventricular drain is likely the most pressing issue and should be addressed first.
  - The drug chart error can be addressed after the external ventricular drain issue is resolved.
  - The acute subdural admission can be prepared for while addressing the other two issues.
- Delegate tasks as appropriate:
  - Address the external ventricular drain issue yourself or delegate to another member of the team, ensuring that the appropriate steps are taken to resolve the issue.
  - Assign someone to re-write the drug chart error.
  - Prepare for the acute subdural admission by ensuring that the necessary equipment, personnel, and resources are available.
- Document the situation and any actions taken.

It’s important to remain calm and prioritize the tasks based on urgency to ensure the best outcome for the patients involved.

(b) Selected participants response

Okay. So in this scenario there are several patient safety issues, and this is a clear clinical prioritization issue. So I'd firstly want to gain more information about each of these issues.

- So the drug error, what is it, what are the current patient’s observations and GCS.
- I'd want to know the same about the EVD. Is this patient EVD dependent or not?
- And finally, I’d want to know whether the subdural has had a trauma call, and the current observations and GCS of the patient.
- I’d also want to know, given this is a workforce issue, if there is anybody else who can potentially support me. Is there another SHO available? Is there a nurse practitioner available? I’m particularly worried about the EVD and the subdural, so if one of them could review one patient whilst I saw the other.

I think in this instance, with the information you’ve given me, that the acute subdural haematoma is my highest priority, because I’m aware this can be a very serious injury. I’d ensure that a trauma call had been made, and then would go to review them.

*This participant scored highly.*

**(1) You quickly amend the drug chart, and are assured that the EVD patient is GCS 15. You then go to see the subdural patient and prepare him for theatre. As you clerk the patient you notice that he has a positive swab result for Covid-19. How would you proceed?**

(a) ChatGPT’s response

In this situation, you should prioritize the patient’s safety and take appropriate measures to minimize the risk of transmission of the virus. Here are some steps you could take:

- Notify the relevant members of the healthcare team, including the consultant and the infection control team, of the positive COVID-19 result.
- Ensure that the patient is isolated and that appropriate personal protective equipment (PPE) is used by all personnel entering the room.
- Consider postponing the surgical procedure if it is not an emergency.
- Ensure that the patient is transported to the operating room using appropriate isolation and PPE precautions.
- Inform the operating team of the positive COVID-19 result and ensure that all appropriate precautions are taken during the procedure.

It’s important to be vigilant and take all necessary precautions to minimize the risk of transmission and protect both the patient and healthcare workers. Documentation of the situation and actions taken is also crucial.

(b) Selected participants response

If the patient needs to go to theatre but has a positive a COVID result, I’d:

- Follow the local protocol for infection control.
- I’d make sure the patient has been put in a side room.
- Make sure its been documented in the notes.
- Inform the theatre time and bed manager to ensure the patient goes to the appropriate bed, and would ensure the theatre team were aware to clean the theatre appropriately following an operation with a COVID positive patient.
- I would also contact ICU to let them know, and to ensure the patient has the correct post-op bed available.
- I’d then continue plans to get the patient to theatre as this infection shouldn’t stop them from having their surgery.’

*This participant scored an average score across the management station.*

So I would let everybody know that he’s positive for COVID, including theatre staff, and particularly the anaesthetic team who will be intubating the patient as they may need extra PPE equipment. After that it just needs to be safely documented in the notes.

*This participant score below average across the management station.*

**(1) The patient is taken to theatre. Your registrar is scrubbed and ready, but you notice she is swaying. When asked if she is okay she mumbles that ‘she is fine’, and that the consultant said it was fine for her to continue.**

(a) ChatGPT’s response

In this situation, you should prioritize the patient’s safety and well-being, and take appropriate measures to ensure that the procedure is performed by a competent and alert healthcare worker. Here are some steps you could take:

- Assess the registrar’s cognitive and physical abilities to perform the procedure safely.
- If there are concerns about their ability to perform the procedure safely, consider relieving them of their duties for the procedure.
- Consult with the consultant or another senior colleague to determine the best course of action.
- If necessary, arrange for another member of the team to take over the procedure.
- Document the situation and any actions taken, including the reason for the registrar’s condition and the assessment of their ability to work safely.

Patient safety should be the top priority in any medical procedure, and it’s important to ensure that the procedure is performed by a competent and alert healthcare worker.

**(b) Selected participants response**

Okay, so the key issue here is that I know that she’s taken a medication that might impair cognitive thought, and that’s a key patient safety issue, and I’d immediately have to take action to prevent them from operation and making a mistake that might harm the patient. What I’d do is:

- Express my concern to my registrar and ask them to step away from the patient.
- The next thing I’d do is speak to my consultant, the one who is already aware of the situation. The consultant might not have been aware that the registrar is swaying, and hence I’d explain this and I’d ask them to come in and assist with the case.

That would be my immediate management. Once this had resolved, I’d probably do some reflection on this case. I’d also, of course, datix and document whats happened given we’ve had to change the course of the operation.

*This participant score highly in the management station.*

Okay, so I’m a little bit worried that she is swaying and about to operate, thus potentially putting the patient at risk. I’d quickly ring the consultant. I would just note my concerns, and clarify with my consultant that they are in fact happy for this to happen. I would speak with the registrar too and voice my concerns that she is not as well as she seems to be. Based on that, I would offer my support and would scrub in with her and assist to the best of my abilities.

*This participant score below average across the management station.*

**(1) You go to call the consultant and the registrar grabs the phone out your hand, tells you to mind your own business, and tells the theatre staff that she is ready to start the operation now. How would you proceed?**

(a) ChatGPT’s response

In this situation, you are facing a challenge to your authority and should respond in a way that prioritizes patient safety and adheres to professional standards. Here are some steps you could take:

- Remain calm and professional, and avoid confrontation with the registrar.
- Reiterate the importance of ensuring that the procedure is performed by a competent and alert healthcare worker.
- Consult with the consultant or another senior colleague to determine the best course of action.
- If necessary, arrange for another member of the team to take over the procedure.
- Document the situation and any actions taken, including the reason for the registrar’s condition and the assessment of their ability to work safely.

It’s important to ensure that patient safety is the top priority and to follow professional standards, even in difficult situations. If necessary, seek guidance from a senior colleague or relevant professional body.

**(b) Selected participants response**

At this point I’ve got a registrar who wants to persist with the operation despite me trying to escalate it.

- I’d try to garner the support of my colleagues around me to prevent the registrar from operating.
- If I felt that that the registrar was not listening to me and I had serious concerns, I would ask the anaesthetist for help and to stay with the patient whilst I contacted somebody senior to help with the operation – this may be calling in the consultant, or finding another senior member who may be present.
- If not, a very last resort, one may wish to call security to escort the registrar away from site. Though this would be a very last resort, after we’d exhausted all other options.
- Id want to, of course, document everything. This is a serious clinical incident, and so at an appropriate time I’d want to do that.

*This participant scored highly across the management station.*

So at this point I’m still really concerned about the patient and my registrar. What I would like to do is:

- Make sure the rest of the theatre staff are aware of my concerns, and speak to the consultant anaesthetist, and see if I can get additional support to de-escalate the situation.
- I would reiterate my concerns to my registrar, using an appropriate tone in a non-confrontational manner.
- I would then get in contact with the on-call consultant, if I weren’t able to get hold of them I’d get hold of the second on-call consultant, and if not I’d escalate up to the clinical director.
- But overall I’d want to make sure that the operation does not proceed by the registrar without appropriate cover’.

*This participant scored an average score across the management station.*

(1) **How do you recognise that you’re stressed?**

(a) ChatGPT’s response

Stress can manifest in different ways, and it’s important to be aware of the signs in order to manage it effectively. Here are some common signs of stress:

- *Physical symptoms*: Headaches, muscle tension, fatigue, changes in appetite or sleep patterns.
- *Emotional symptoms*: Anxiety, irritability, sadness, mood swings, loss of interest in activities.
- *Cognitive symptoms*: Forgetfulness, difficulty concentrating, indecisiveness, negative thinking.
- *Behavioral symptoms*: Increased substance use, social withdrawal, decreased work performance, changes in eating or sleeping habits.
- *Psychosomatic symptoms*: Digestive problems, rapid heartbeat, skin problems.

It’s important to pay attention to any changes in your physical, emotional, cognitive, behavioral, or psychosomatic symptoms and seek support if necessary. Regular self-care and stress management activities, such as exercise, relaxation techniques, and healthy coping mechanisms, can also help prevent and manage stress.

(b) Selected participants response

One of the ways that I recognise that I’m stressed is that I become a bit more irritable. I might snap at colleagues or seniors, and I can find that I’m less efficient with my time. I think that when you recognise that you’re stressed its important to have a good coping strategies. First, its important to have good support from your colleagues and your seniors to manage your workload. Longer term, its important to utilise coping mechanisms. For me, that’s engaging in my hobbies such as [redacted to maintain anonymity].

*This participant scored highly.*
